# (4′-All­yloxy-2,2′:6′,2′′-terpyridine)(dibenzoyl­methanido)dinitratoerbium(III) acetonitrile solvate

**DOI:** 10.1107/S1600536809055032

**Published:** 2010-01-09

**Authors:** Qunbo Mei, Bihai Tong

**Affiliations:** a Jiangsu Key Laboratory of Organic Electronics & Information Displays and, Institute of Advanced Materials (IAM), Nanjing University of Posts & Telecommunications, Nanjing 210046, People’s Republic of China; b Institute of Molecular Engineering & Applied Chemistry, College of Metallurgy and Resources, Anhui University of Technology, Maanshan 243002, People’s Republic of China

## Abstract

The title complex, [Er(C_15_H_11_O_2_)(NO_3_)_2_(C_18_H_15_N_3_O)]·CH_3_CN, has been synthesized from 4′-all­yloxy-2,2′:6′,2′′-terpyridine (altpy), dibenzoyl­methane and erbium nitrate. The distorted monocapped square anti­prismatic coordination polyhedron is formed by a bidentate dibenzoyl­methanide residue, a tridentate altpy ligand and two nitrate anions that act as bidentate ligands and occupy mutually *trans* sites.

## Related literature

For the use of lanthanide complexes as functional materials, see: Sun *et al.* (2005 ▶). For antenna effects, see: Sabbatini *et al.* (1993 ▶). For related structures, see: Niu *et al.* (1997 ▶); Neelgund *et al.* (2007 ▶); Fukuda *et al.* (2002 ▶); Hunter *et al.* (2007 ▶).
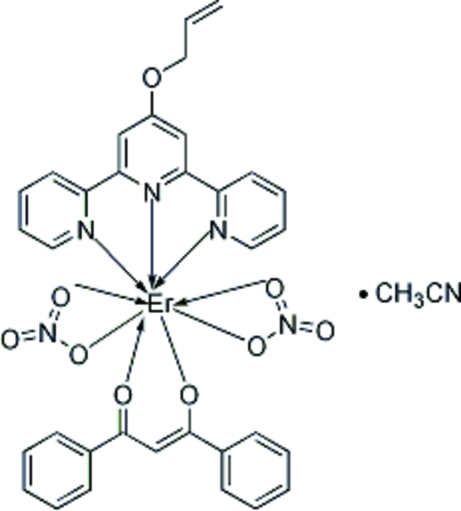


## Experimental

### 

#### Crystal data

[Er(C_15_H_11_O_2_)(NO_3_)_2_(C_18_H_15_N_3_O)]·C_2_H_3_N
*M* *_r_* = 844.90
Monoclinic, 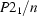

*a* = 13.245 (4) Å
*b* = 15.871 (4) Å
*c* = 16.135 (5) Å
β = 103.374 (6)°
*V* = 3299.8 (16) Å^3^
*Z* = 4
Mo *K*α radiation
μ = 2.61 mm^−1^
*T* = 173 K
0.26 × 0.24 × 0.22 mm

#### Data collection

Bruker SMART CCD area-detector diffractometer
Absorption correction: multi-scan (*SADABS*; Bruker, 1997 ▶) *T*_min_ = 0.550, *T*_max_ = 0.598
15598 measured reflections
6429 independent reflections
4383 reflections with *I* > 2σ(*I*)
*R*_int_ = 0.052

#### Refinement

*R*[*F* ^2^ > 2σ(*F* ^2^)] = 0.036
*wR*(*F* ^2^) = 0.088
*S* = 1.04
6429 reflections
460 parameters
H-atom parameters constrained
Δρ_max_ = 1.65 e Å^−3^
Δρ_min_ = −1.14 e Å^−3^

### 

Data collection: *SMART* (Bruker, 1997 ▶); cell refinement: *SAINT* (Bruker, 1997 ▶); data reduction: *SAINT*; program(s) used to solve structure: *SHELXS97* (Sheldrick, 2008 ▶); program(s) used to refine structure: *SHELXL97* (Sheldrick, 2008 ▶); molecular graphics: *SHELXTL* (Sheldrick, 2008 ▶); software used to prepare material for publication: *SHELXTL*.

## Supplementary Material

Crystal structure: contains datablocks I, global. DOI: 10.1107/S1600536809055032/si2232sup1.cif

Structure factors: contains datablocks I. DOI: 10.1107/S1600536809055032/si2232Isup2.hkl

Additional supplementary materials:  crystallographic information; 3D view; checkCIF report

## Figures and Tables

**Table 1:** Selected bond lengths (Å)

Er1—O3	2.224 (3)
Er1—O2	2.228 (4)
Er1—O4	2.410 (4)
Er1—O9	2.425 (4)
Er1—N2	2.447 (4)
Er1—N3	2.460 (4)
Er1—O5	2.465 (4)
Er1—O8	2.468 (4)
Er1—N1	2.515 (4)

**Fig. 1.**
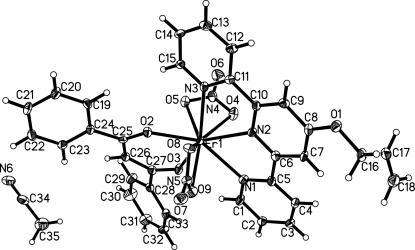
The molecular structure of (I), with atom labels and 30% probability displacement ellipsoids for non-H atoms.

## supplementary crystallographic information

### Comment

Recently, much attention has been paid to near-infrared (NIR) luminescence of trivalent lanthanide ions such as erbium (Er^3+^) and neodymium (Nd^3+^), because they show luminescence in the telecommunication low-loss NIR-regions of silica (Sun *et al.*, (2005)). However, it is difficult to generate this luminescence by direct excitation of these NIR-luminescence lanthanide ions due to some quenching effects as well as their poor absorption abilities. A method to avoid quenching of the excited state is to shield the lanthanide ion from the deactivating groups by a shell of organic ligands. Another benefit of using organic ligands is that energy absorbed by a ligand containing a chromophoric group, can be transferred to the lanthanide ion. This mechanism is called the antenna effect (Sabbatini *et al.*, (1993)). In the title compound, [Er(altpy)(dbm)(NO_3_)_2_].CH_3_CN(altpy=4'-allyloxy-2, 2':6', 2''-terpyridine, dbm=dibenzoylmethanate), each Er(III) atom is in a nine coordinate environment comprising two oxygen atoms from the bidentate dbm ligand, three nitrogen atoms from the tridentate altpy ligand and four oxygen atoms from two tertiary nitrate anions that act as bidentate ligands and occupy mutually *trans* sites in the coordination polyhedron. The coordination polyhedron is a distorted monocapped square antiprism. The Er—O distances lie in two groups, those to the beta-diketone oxygen atoms in the range 2.224 (3)–2.228 (4) Å and those to nitrate O atoms in the range 2.410 (4)–2.468 (4) Å. These are comparable to those [2.485 (19), 2.600 (15) Å] in the nine-coordinate complex [Er_2_(O_2_CMe)_4_(NO_3_)_2_(phen)_2_] (phen=1,10-phenanthroline) which also contains bidentate chelating nitrate anions (Niu *et al.*, 1997). The O—Er—O angle (76.97 (13) °) of the beta-diketonate ligand is somewhat higher as compared to those found in the erbium tris(beta-diketonates) type of complexes (73.65 (49) °) (Neelgund *et al.*, (2007)). The average Er—N distance (2.474 (4) Å) is slightly shorter than that in the nine-coordinate complex [Er(terpy) (acac) (NO_3_)_2_^-^] (2.503 (4) Å)(Fukuda *et al.*, (2002)). The geometrical parameters of the [NO_3_]^-^ anions in the title complex are as expected with normal distances and angles, comparable to those reported by Hunter *et al.*, (2007) for a complex also containing bidentate chelating nitrate anions.

### Experimental

The title compound was obtained by refluxing erbium nitrate, 4'-allyloxy-2, 2':6',2''-terpyridine and dibenzoylmethanate in ethanol to give the title compound as a yellow precipitate in 81% yield. Recrystallization from ethanol and acetonitrile (1:1) gave yellow block-like crystals suitable for an X-ray diffraction determination. Anal.Calcd. for C_35_H_29_ErN_6_O_9_: C, 52.60, H, 3.46, N, 9.95%. Found:*C*, 51.70, H, 3.71, N, 9.87%.

### Refinement

H atoms were positioned geometrically and refined using a riding model (including free rotation about the ethanol C—C bond), with C—H = 0.95–0.99 Å and with *U*_iso_(H) = 1.2 (1.5 for methyl groups) *U*_eq_(C).

### Crystal data

**Table:**

[Er(C_15_H_11_O_2_)(NO_3_)_2_(C_18_H_15_N_3_O)]·C_2_H_3_N	*F*(000) = 1684
*M**_r_* = 844.90	*D*_x_ = 1.701 Mg m^−^^3^
Monoclinic, *P*2_1_/*n*	Mo *K*α radiation, λ = 0.71073 Å
Hall symbol: -P 2yn	Cell parameters from 4650 reflections
*a* = 13.245 (4) Å	θ = 2.6–26.6°
*b* = 15.871 (4) Å	µ = 2.61 mm^−^^1^
*c* = 16.135 (5) Å	*T* = 173 K
β = 103.374 (6)°	Block, yellow
*V* = 3299.8 (16) Å^3^	0.26 × 0.24 × 0.22 mm
*Z* = 4	

### Data collection

**Table:**

Bruker SMART CCD area-detector diffractometer	6429 independent reflections
Radiation source: fine-focus sealed tube	4383 reflections with *I* > 2σ(*I*)
graphite	*R*_int_ = 0.052
φ and ω scans	θ_max_ = 26.0°, θ_min_ = 1.8°
Absorption correction: multi-scan (*SADABS*; Bruker, 1997)	*h* = −11→16
*T*_min_ = 0.550, *T*_max_ = 0.598	*k* = −19→19
15598 measured reflections	*l* = −19→15

### Refinement

**Table:**

Refinement on *F*^2^	Primary atom site location: structure-invariant direct methods
Least-squares matrix: full	Secondary atom site location: difference Fourier map
*R*[*F*^2^ > 2σ(*F*^2^)] = 0.036	Hydrogen site location: inferred from neighbouring sites
*wR*(*F*^2^) = 0.088	H-atom parameters constrained
*S* = 1.04	*w* = 1/[σ^2^(*F*_o_^2^) + (0.0365*P*)^2^] where *P* = (*F*_o_^2^ + 2*F*_c_^2^)/3
6429 reflections	(Δ/σ)_max_ = 0.002
460 parameters	Δρ_max_ = 1.65 e Å^−^^3^
0 restraints	Δρ_min_ = −1.14 e Å^−^^3^

### Special details

**Table:**

Geometry. All e.s.d.'s (except the e.s.d. in the dihedral angle between two l.s. planes) are estimated using the full covariance matrix. The cell e.s.d.'s are taken into account individually in the estimation of e.s.d.'s in distances, angles and torsion angles; correlations between e.s.d.'s in cell parameters are only used when they are defined by crystal symmetry. An approximate (isotropic) treatment of cell e.s.d.'s is used for estimating e.s.d.'s involving l.s. planes.
Refinement. Refinement of *F*^2^ against ALL reflections. The weighted *R*-factor *wR* and goodness of fit *S* are based on *F*^2^, conventional *R*-factors *R* are based on *F*, with *F* set to zero for negative *F*^2^. The threshold expression of *F*^2^ > σ(*F*^2^) is used only for calculating *R*-factors(gt) *etc*. and is not relevant to the choice of reflections for refinement. *R*-factors based on *F*^2^ are statistically about twice as large as those based on *F*, and *R*- factors based on ALL data will be even larger.

### Fractional atomic coordinates and isotropic or equivalent isotropic displacement parameters (Å^2^)

**Table:**

	*x*	*y*	*z*	*U*_iso_*/*U*_eq_	
Er1	0.756234 (17)	0.801711 (15)	0.037240 (16)	0.01847 (8)	
O2	0.6625 (3)	0.6940 (2)	0.0666 (2)	0.0255 (9)	
O9	0.7831 (3)	0.8367 (3)	0.1870 (3)	0.0384 (11)	
N5	0.6926 (4)	0.8638 (3)	0.1844 (3)	0.0277 (12)	
O3	0.8761 (3)	0.7066 (2)	0.0928 (2)	0.0278 (9)	
O8	0.6304 (3)	0.8630 (3)	0.1117 (2)	0.0306 (10)	
N2	0.7337 (3)	0.9511 (3)	0.0000 (3)	0.0227 (10)	
C13	0.4028 (4)	0.8715 (4)	−0.1717 (4)	0.0326 (15)	
H13A	0.3365	0.8842	−0.2069	0.039*	
N1	0.9170 (3)	0.8903 (3)	0.0826 (3)	0.0257 (11)	
N3	0.5943 (3)	0.8339 (3)	−0.0678 (3)	0.0244 (11)	
C26	0.7835 (4)	0.5834 (4)	0.1087 (3)	0.0235 (13)	
H26	0.7901	0.5246	0.1200	0.028*	
C24	0.5947 (4)	0.5659 (3)	0.1060 (4)	0.0240 (13)	
C6	0.8061 (4)	1.0085 (4)	0.0366 (4)	0.0244 (13)	
C20	0.4118 (4)	0.5409 (4)	0.0781 (4)	0.0333 (15)	
H20A	0.3431	0.5574	0.0511	0.040*	
C23	0.6107 (4)	0.4949 (4)	0.1589 (4)	0.0280 (14)	
H23A	0.6792	0.4786	0.1864	0.034*	
O7	0.6658 (3)	0.8903 (3)	0.2473 (3)	0.0425 (12)	
C1	1.0107 (4)	0.8569 (4)	0.1162 (4)	0.0296 (14)	
H1A	1.0168	0.7973	0.1201	0.035*	
C18	0.9133 (5)	1.2961 (4)	0.0249 (4)	0.0433 (16)	
H18A	0.9352	1.2855	0.0843	0.052*	
H18B	0.9627	1.3111	−0.0068	0.052*	
C33	1.0601 (4)	0.6243 (4)	0.1839 (4)	0.0282 (14)	
H33A	1.0537	0.6808	0.2013	0.034*	
C27	0.8725 (4)	0.6284 (4)	0.1095 (4)	0.0254 (13)	
C25	0.6834 (4)	0.6185 (4)	0.0921 (3)	0.0233 (13)	
C4	0.9941 (4)	1.0266 (4)	0.1053 (4)	0.0290 (14)	
H4A	0.9867	1.0861	0.1012	0.035*	
C3	1.0896 (4)	0.9908 (4)	0.1391 (3)	0.0284 (14)	
H3A	1.1490	1.0255	0.1579	0.034*	
C19	0.4941 (4)	0.5886 (4)	0.0659 (3)	0.0272 (14)	
H19A	0.4821	0.6368	0.0301	0.033*	
C5	0.9095 (4)	0.9748 (3)	0.0775 (4)	0.0246 (13)	
C28	0.9739 (4)	0.5836 (4)	0.1333 (4)	0.0252 (13)	
C11	0.5651 (4)	0.9147 (4)	−0.0845 (3)	0.0228 (13)	
C22	0.5273 (4)	0.4488 (4)	0.1709 (4)	0.0326 (15)	
H22A	0.5386	0.4020	0.2087	0.039*	
C14	0.4322 (4)	0.7896 (4)	−0.1555 (3)	0.0283 (14)	
H14A	0.3874	0.7448	−0.1798	0.034*	
C15	0.5281 (4)	0.7731 (4)	−0.1033 (3)	0.0247 (13)	
H15A	0.5482	0.7161	−0.0920	0.030*	
C29	0.9859 (4)	0.5007 (4)	0.1094 (4)	0.0371 (16)	
H29A	0.9283	0.4713	0.0758	0.044*	
C10	0.6405 (4)	0.9802 (3)	−0.0439 (3)	0.0244 (13)	
C12	0.4691 (4)	0.9361 (4)	−0.1370 (4)	0.0294 (14)	
H12A	0.4500	0.9933	−0.1486	0.035*	
C9	0.6173 (4)	1.0647 (4)	−0.0499 (3)	0.0260 (14)	
H9A	0.5514	1.0830	−0.0818	0.031*	
C7	0.7868 (4)	1.0951 (3)	0.0333 (4)	0.0262 (13)	
H7A	0.8392	1.1336	0.0600	0.031*	
C17	0.8142 (5)	1.2901 (4)	−0.0134 (4)	0.0371 (16)	
H17A	0.7946	1.3011	−0.0729	0.045*	
C8	0.6904 (4)	1.1238 (4)	−0.0091 (4)	0.0277 (14)	
O1	0.6601 (3)	1.2051 (2)	−0.0152 (3)	0.0344 (10)	
C32	1.1545 (4)	0.5835 (4)	0.2091 (4)	0.0345 (15)	
H32A	1.2123	0.6120	0.2435	0.041*	
C21	0.4271 (4)	0.4697 (4)	0.1288 (4)	0.0329 (15)	
H21A	0.3700	0.4359	0.1346	0.040*	
C31	1.1647 (5)	0.5019 (4)	0.1844 (4)	0.0431 (17)	
H31A	1.2294	0.4737	0.2017	0.052*	
C34	0.9374 (6)	0.2095 (5)	0.2523 (4)	0.0456 (18)	
C35	1.0307 (5)	0.2525 (5)	0.2958 (5)	0.064 (2)	
H35A	1.0281	0.3112	0.2767	0.096*	
H35B	1.0912	0.2246	0.2828	0.096*	
H35C	1.0361	0.2508	0.3574	0.096*	
N6	0.8619 (6)	0.1790 (4)	0.2183 (4)	0.069 (2)	
O5	0.7447 (3)	0.6999 (3)	−0.0800 (3)	0.0328 (9)	
O4	0.8224 (3)	0.8173 (3)	−0.0890 (3)	0.0379 (11)	
N4	0.7853 (4)	0.7484 (3)	−0.1245 (3)	0.0319 (12)	
C2	1.0982 (4)	0.9046 (4)	0.1452 (4)	0.0293 (14)	
H2A	1.1631	0.8787	0.1690	0.035*	
C16	0.7312 (4)	1.2670 (4)	0.0308 (4)	0.0345 (16)	
H16A	0.7636	1.2446	0.0880	0.041*	
H16B	0.6919	1.3184	0.0386	0.041*	
C30	1.0801 (5)	0.4608 (4)	0.1338 (5)	0.051 (2)	
H30A	1.0874	0.4046	0.1159	0.061*	
O6	0.7867 (4)	0.7318 (3)	−0.1977 (3)	0.0538 (13)	

### Atomic displacement parameters (Å^2^)

**Table:**

	*U*^11^	*U*^22^	*U*^33^	*U*^12^	*U*^13^	*U*^23^
Er1	0.01372 (12)	0.01403 (13)	0.02633 (15)	0.00049 (12)	0.00190 (9)	0.00175 (13)
O2	0.0199 (19)	0.022 (2)	0.034 (2)	0.0043 (18)	0.0059 (16)	0.0071 (19)
O9	0.024 (2)	0.043 (3)	0.046 (3)	0.005 (2)	0.0030 (19)	−0.002 (2)
N5	0.026 (3)	0.029 (3)	0.026 (3)	−0.003 (2)	0.001 (2)	−0.001 (2)
O3	0.020 (2)	0.018 (2)	0.045 (3)	0.0015 (17)	0.0047 (17)	0.0048 (19)
O8	0.029 (2)	0.037 (3)	0.024 (2)	0.0065 (19)	0.0013 (18)	0.0023 (19)
N2	0.018 (2)	0.020 (3)	0.029 (3)	0.000 (2)	0.0032 (19)	0.000 (2)
C13	0.023 (3)	0.044 (4)	0.029 (4)	0.005 (3)	0.003 (3)	0.002 (3)
N1	0.020 (3)	0.025 (3)	0.031 (3)	0.003 (2)	0.004 (2)	−0.001 (2)
N3	0.026 (3)	0.019 (3)	0.029 (3)	−0.001 (2)	0.007 (2)	0.001 (2)
C26	0.019 (3)	0.020 (3)	0.031 (4)	0.005 (2)	0.004 (2)	0.005 (3)
C24	0.021 (3)	0.022 (3)	0.030 (3)	−0.002 (2)	0.008 (2)	0.002 (3)
C6	0.013 (3)	0.026 (3)	0.034 (4)	0.004 (2)	0.006 (2)	0.006 (3)
C20	0.023 (3)	0.026 (4)	0.050 (4)	−0.001 (3)	0.006 (3)	0.004 (3)
C23	0.023 (3)	0.029 (4)	0.029 (4)	−0.002 (3)	0.000 (3)	0.003 (3)
O7	0.042 (3)	0.052 (3)	0.036 (3)	−0.006 (2)	0.014 (2)	−0.014 (2)
C1	0.019 (3)	0.020 (3)	0.048 (4)	0.000 (3)	0.004 (3)	0.000 (3)
C18	0.048 (4)	0.040 (4)	0.044 (4)	−0.009 (4)	0.015 (3)	−0.003 (3)
C33	0.024 (3)	0.028 (3)	0.035 (4)	0.000 (3)	0.011 (3)	0.004 (3)
C27	0.024 (3)	0.023 (3)	0.029 (3)	0.003 (3)	0.006 (3)	0.002 (3)
C25	0.019 (3)	0.029 (3)	0.018 (3)	−0.003 (2)	−0.003 (2)	0.001 (3)
C4	0.031 (3)	0.022 (3)	0.033 (4)	−0.006 (3)	0.004 (3)	0.001 (3)
C3	0.024 (3)	0.031 (4)	0.027 (4)	−0.008 (3)	0.000 (3)	−0.003 (3)
C19	0.025 (3)	0.028 (3)	0.028 (3)	0.003 (3)	0.005 (3)	0.010 (3)
C5	0.021 (3)	0.020 (3)	0.033 (4)	0.002 (2)	0.005 (3)	0.004 (3)
C28	0.018 (3)	0.027 (3)	0.030 (4)	0.004 (3)	0.005 (2)	0.009 (3)
C11	0.020 (3)	0.027 (3)	0.022 (3)	0.001 (3)	0.005 (2)	−0.003 (3)
C22	0.037 (4)	0.028 (4)	0.034 (4)	−0.002 (3)	0.010 (3)	0.008 (3)
C14	0.022 (3)	0.037 (4)	0.023 (3)	−0.006 (3)	0.000 (2)	−0.006 (3)
C15	0.027 (3)	0.022 (3)	0.024 (3)	−0.003 (2)	0.002 (3)	−0.002 (2)
C29	0.025 (3)	0.030 (4)	0.052 (4)	0.002 (3)	0.001 (3)	−0.004 (3)
C10	0.023 (3)	0.023 (3)	0.027 (3)	−0.002 (2)	0.006 (2)	0.003 (3)
C12	0.023 (3)	0.033 (4)	0.031 (4)	0.006 (3)	0.002 (3)	−0.004 (3)
C9	0.022 (3)	0.027 (3)	0.028 (3)	0.006 (3)	0.002 (2)	0.002 (3)
C7	0.025 (3)	0.015 (3)	0.038 (4)	−0.005 (2)	0.006 (3)	−0.001 (3)
C17	0.050 (4)	0.027 (4)	0.033 (4)	−0.001 (3)	0.006 (3)	0.003 (3)
C8	0.030 (3)	0.020 (3)	0.034 (4)	−0.001 (3)	0.010 (3)	0.002 (3)
O1	0.031 (2)	0.020 (2)	0.047 (3)	0.0040 (19)	−0.0023 (19)	0.001 (2)
C32	0.017 (3)	0.045 (4)	0.036 (4)	−0.001 (3)	−0.005 (3)	0.002 (3)
C21	0.026 (3)	0.030 (4)	0.047 (4)	−0.009 (3)	0.017 (3)	−0.003 (3)
C31	0.027 (4)	0.043 (4)	0.055 (5)	0.016 (3)	0.003 (3)	−0.001 (4)
C34	0.058 (5)	0.048 (5)	0.027 (4)	0.005 (4)	0.003 (3)	0.001 (3)
C35	0.047 (5)	0.084 (6)	0.062 (6)	−0.002 (5)	0.015 (4)	−0.008 (5)
N6	0.083 (5)	0.072 (5)	0.038 (4)	−0.017 (4)	−0.012 (4)	0.001 (3)
O5	0.034 (2)	0.027 (2)	0.039 (2)	−0.004 (2)	0.0119 (18)	0.001 (2)
O4	0.039 (3)	0.029 (3)	0.051 (3)	−0.005 (2)	0.021 (2)	−0.001 (2)
N4	0.037 (3)	0.020 (3)	0.039 (4)	0.006 (2)	0.011 (3)	0.001 (3)
C2	0.016 (3)	0.030 (4)	0.037 (4)	0.000 (3)	−0.003 (3)	0.005 (3)
C16	0.040 (4)	0.014 (3)	0.047 (4)	0.006 (3)	0.005 (3)	0.002 (3)
C30	0.041 (4)	0.039 (4)	0.068 (5)	0.017 (3)	0.004 (4)	−0.012 (4)
O6	0.083 (4)	0.049 (3)	0.036 (3)	0.004 (3)	0.026 (3)	−0.008 (2)

### Geometric parameters (Å, °)

**Table:**

Er1—O3	2.224 (3)	C33—H33A	0.9500
Er1—O2	2.228 (4)	C27—C28	1.490 (7)
Er1—O4	2.410 (4)	C4—C5	1.378 (7)
Er1—O9	2.425 (4)	C4—C3	1.379 (7)
Er1—N2	2.447 (4)	C4—H4A	0.9500
Er1—N3	2.460 (4)	C3—C2	1.375 (8)
Er1—O5	2.465 (4)	C3—H3A	0.9500
Er1—O8	2.468 (4)	C19—H19A	0.9500
Er1—N1	2.515 (4)	C28—C29	1.390 (8)
Er1—N4	2.853 (6)	C11—C12	1.397 (7)
Er1—N5	2.872 (5)	C11—C10	1.486 (7)
O2—C25	1.275 (6)	C22—C21	1.385 (7)
O9—N5	1.265 (6)	C22—H22A	0.9500
N5—O7	1.224 (6)	C14—C15	1.377 (7)
N5—O8	1.268 (5)	C14—H14A	0.9500
O3—C27	1.274 (6)	C15—H15A	0.9500
N2—C10	1.355 (6)	C29—C30	1.372 (8)
N2—C6	1.355 (7)	C29—H29A	0.9500
C13—C14	1.365 (8)	C10—C9	1.373 (8)
C13—C12	1.381 (8)	C12—H12A	0.9500
C13—H13A	0.9500	C9—C8	1.399 (7)
N1—C1	1.343 (6)	C9—H9A	0.9500
N1—C5	1.347 (7)	C7—C8	1.379 (7)
N3—C15	1.340 (7)	C7—H7A	0.9500
N3—C11	1.348 (7)	C17—C16	1.488 (8)
C26—C27	1.376 (7)	C17—H17A	0.9500
C26—C25	1.406 (7)	C8—O1	1.349 (6)
C26—H26	0.9500	O1—C16	1.442 (7)
C24—C19	1.387 (7)	C32—C31	1.370 (9)
C24—C23	1.398 (7)	C32—H32A	0.9500
C24—C25	1.501 (7)	C21—H21A	0.9500
C6—C7	1.396 (7)	C31—C30	1.387 (9)
C6—C5	1.476 (7)	C31—H31A	0.9500
C20—C19	1.378 (7)	C34—N6	1.132 (8)
C20—C21	1.382 (8)	C34—C35	1.443 (10)
C20—H20A	0.9500	C35—H35A	0.9800
C23—C22	1.376 (7)	C35—H35B	0.9800
C23—H23A	0.9500	C35—H35C	0.9800
C1—C2	1.373 (7)	O5—N4	1.256 (6)
C1—H1A	0.9500	O4—N4	1.279 (6)
C18—C17	1.318 (8)	N4—O6	1.214 (6)
C18—H18A	0.9500	C2—H2A	0.9500
C18—H18B	0.9500	C16—H16A	0.9900
C33—C32	1.383 (7)	C16—H16B	0.9900
C33—C28	1.399 (7)	C30—H30A	0.9500
			
O3—Er1—O2	76.97 (13)	C17—C18—H18B	120.0
O3—Er1—O4	92.59 (14)	H18A—C18—H18B	120.0
O2—Er1—O4	126.04 (14)	C32—C33—C28	121.1 (6)
O3—Er1—O9	80.05 (14)	C32—C33—H33A	119.5
O2—Er1—O9	85.66 (14)	C28—C33—H33A	119.5
O4—Er1—O9	145.20 (14)	O3—C27—C26	125.4 (5)
O3—Er1—N2	142.34 (13)	O3—C27—C28	116.3 (5)
O2—Er1—N2	138.91 (13)	C26—C27—C28	118.3 (5)
O4—Er1—N2	74.94 (14)	O2—C25—C26	124.0 (5)
O9—Er1—N2	90.25 (15)	O2—C25—C24	116.9 (5)
O3—Er1—N3	147.82 (14)	C26—C25—C24	119.1 (5)
O2—Er1—N3	82.30 (14)	C5—C4—C3	119.1 (5)
O4—Er1—N3	79.97 (15)	C5—C4—H4A	120.5
O9—Er1—N3	122.85 (14)	C3—C4—H4A	120.5
N2—Er1—N3	65.93 (14)	C2—C3—C4	119.4 (5)
O3—Er1—O5	77.08 (14)	C2—C3—H3A	120.3
O2—Er1—O5	73.83 (13)	C4—C3—H3A	120.3
O4—Er1—O5	52.32 (13)	C20—C19—C24	119.8 (5)
O9—Er1—O5	152.10 (14)	C20—C19—H19A	120.1
N2—Er1—O5	117.65 (14)	C24—C19—H19A	120.1
N3—Er1—O5	73.64 (14)	N1—C5—C4	122.0 (5)
O3—Er1—O8	124.80 (13)	N1—C5—C6	115.9 (5)
O2—Er1—O8	74.14 (13)	C4—C5—C6	122.0 (5)
O4—Er1—O8	142.14 (13)	C29—C28—C33	117.9 (5)
O9—Er1—O8	52.00 (13)	C29—C28—C27	122.4 (5)
N2—Er1—O8	71.35 (14)	C33—C28—C27	119.7 (5)
N3—Er1—O8	70.99 (14)	N3—C11—C12	122.1 (5)
O5—Er1—O8	134.65 (13)	N3—C11—C10	116.4 (5)
O3—Er1—N1	77.54 (14)	C12—C11—C10	121.5 (5)
O2—Er1—N1	147.48 (14)	C23—C22—C21	120.9 (6)
O4—Er1—N1	74.91 (14)	C23—C22—H22A	119.6
O9—Er1—N1	70.29 (14)	C21—C22—H22A	119.6
N2—Er1—N1	64.97 (14)	C13—C14—C15	118.7 (5)
N3—Er1—N1	128.97 (15)	C13—C14—H14A	120.7
O5—Er1—N1	119.19 (14)	C15—C14—H14A	120.7
O8—Er1—N1	105.04 (14)	N3—C15—C14	123.0 (5)
O3—Er1—N4	86.12 (14)	N3—C15—H15A	118.5
O2—Er1—N4	99.60 (14)	C14—C15—H15A	118.5
O4—Er1—N4	26.44 (13)	C30—C29—C28	120.8 (6)
O9—Er1—N4	163.74 (14)	C30—C29—H29A	119.6
N2—Er1—N4	95.58 (15)	C28—C29—H29A	119.6
N3—Er1—N4	73.29 (14)	N2—C10—C9	122.1 (5)
O5—Er1—N4	26.02 (13)	N2—C10—C11	115.5 (5)
O8—Er1—N4	144.24 (13)	C9—C10—C11	122.4 (5)
N1—Er1—N4	98.48 (15)	C13—C12—C11	118.0 (6)
O3—Er1—N5	102.61 (14)	C13—C12—H12A	121.0
O2—Er1—N5	78.62 (13)	C11—C12—H12A	121.0
O4—Er1—N5	153.86 (14)	C10—C9—C8	120.1 (5)
O9—Er1—N5	25.92 (12)	C10—C9—H9A	119.9
N2—Er1—N5	80.12 (14)	C8—C9—H9A	119.9
N3—Er1—N5	97.02 (14)	C8—C7—C6	119.0 (5)
O5—Er1—N5	151.79 (13)	C8—C7—H7A	120.5
O8—Er1—N5	26.08 (12)	C6—C7—H7A	120.5
N1—Er1—N5	87.62 (14)	C18—C17—C16	123.9 (6)
N4—Er1—N5	170.31 (13)	C18—C17—H17A	118.0
C25—O2—Er1	134.4 (3)	C16—C17—H17A	118.0
N5—O9—Er1	97.2 (3)	O1—C8—C7	125.3 (5)
O7—N5—O9	122.9 (5)	O1—C8—C9	116.4 (5)
O7—N5—O8	121.3 (5)	C7—C8—C9	118.2 (5)
O9—N5—O8	115.7 (5)	C8—O1—C16	117.8 (4)
O7—N5—Er1	179.8 (4)	C31—C32—C33	120.0 (6)
O9—N5—Er1	56.9 (3)	C31—C32—H32A	120.0
O8—N5—Er1	58.9 (3)	C33—C32—H32A	120.0
C27—O3—Er1	133.5 (3)	C20—C21—C22	118.6 (5)
N5—O8—Er1	95.0 (3)	C20—C21—H21A	120.7
C10—N2—C6	117.8 (5)	C22—C21—H21A	120.7
C10—N2—Er1	120.2 (3)	C32—C31—C30	119.7 (6)
C6—N2—Er1	120.7 (3)	C32—C31—H31A	120.2
C14—C13—C12	120.2 (5)	C30—C31—H31A	120.2
C14—C13—H13A	119.9	N6—C34—C35	176.9 (9)
C12—C13—H13A	119.9	C34—C35—H35A	109.5
C1—N1—C5	117.8 (5)	C34—C35—H35B	109.5
C1—N1—Er1	122.6 (4)	H35A—C35—H35B	109.5
C5—N1—Er1	119.5 (3)	C34—C35—H35C	109.5
C15—N3—C11	118.0 (5)	H35A—C35—H35C	109.5
C15—N3—Er1	121.7 (4)	H35B—C35—H35C	109.5
C11—N3—Er1	120.0 (3)	N4—O5—Er1	94.5 (3)
C27—C26—C25	124.3 (5)	N4—O4—Er1	96.5 (3)
C27—C26—H26	117.9	O6—N4—O5	121.9 (5)
C25—C26—H26	117.9	O6—N4—O4	122.0 (5)
C19—C24—C23	119.2 (5)	O5—N4—O4	116.0 (5)
C19—C24—C25	119.0 (5)	O6—N4—Er1	171.8 (4)
C23—C24—C25	121.7 (5)	O5—N4—Er1	59.5 (3)
N2—C6—C7	122.7 (5)	O4—N4—Er1	57.0 (3)
N2—C6—C5	116.2 (5)	C1—C2—C3	118.4 (5)
C7—C6—C5	121.0 (5)	C1—C2—H2A	120.8
C19—C20—C21	121.4 (5)	C3—C2—H2A	120.8
C19—C20—H20A	119.3	O1—C16—C17	112.8 (5)
C21—C20—H20A	119.3	O1—C16—H16A	109.0
C22—C23—C24	120.0 (5)	C17—C16—H16A	109.0
C22—C23—H23A	120.0	O1—C16—H16B	109.0
C24—C23—H23A	120.0	C17—C16—H16B	109.0
N1—C1—C2	123.2 (5)	H16A—C16—H16B	107.8
N1—C1—H1A	118.4	C29—C30—C31	120.6 (6)
C2—C1—H1A	118.4	C29—C30—H30A	119.7
C17—C18—H18A	120.0	C31—C30—H30A	119.7
